# Evaluation of shear bond strength based on substructure materials and ceramic veneering techniques

**DOI:** 10.1111/jopr.13889

**Published:** 2024-05-31

**Authors:** Han‐Sol Song, Yoon‐Hyuk Huh, Chan‐Jin Park, Lee‐Ra Cho, Kyung‐Ho Ko

**Affiliations:** ^1^ Department of Prosthodontics and Research Institute of Oral Science College of Dentistry Gangneung‐Wonju National University Gangneung Republic of Korea

**Keywords:** cobalt‐chrome alloy, layering technique, pressing technique, shear bond strength, zirconia

## Abstract

**Purpose:**

Bilayered restorations have both the strength of the substructure material and the esthetics of the veneer material; however, they should have appropriate bonding between the two materials. This study aimed to evaluate the shear bond strength (SBS) according to the substructure material and veneering technique used in bilayered restorations.

**Materials and Methods:**

The experimental group was divided into four groups (*n* = 15 per group) based on the substructure materials (cobalt‐chromium [Co‐Cr] alloy and 3 mol% yttrium‐stabilized tetragonal zirconia polycrystal [3Y‐TZP]) and veneering techniques (pressing and layering). Veneering was performed with disk shape (diameter: 5 mm, height: 2 mm) on a substructure using each veneering technique. Shear stress was applied to the interface of the substructure and the veneering ceramic using a universal testing machine. The shear bond strength, according to the substructure and veneering technique, was analyzed using a two‐way analysis of variance with a post‐hoc Tukey's honestly significant difference test. The failure mode was observed, and the surface was analyzed using a scanning electron microscope and energy‐dispersive spectroscopy.

**Results:**

The shSBS of the Co‐Cr alloy and 3Y‐TZP substructure was not different (*p* > 0.05); however, the pressing technique showed a higher SBS than the layering technique (*p* < 0.05). The SBS did not differ depending on the veneering technique in the Co‐Cr alloys (*p* > 0.05), whereas the SBS in the pressing technique was higher than that in the layering technique for 3Y‐TZP (*p* < 0.05). In the layering technique, the Co‐Cr alloy showed a higher SBS than 3Y‐TZP (*p* < 0.05). In the failure mode, mixed failure occurred most frequently in all groups. Extensive elemental interdiffusion was observed through the opaque layer in the Co‐Cr alloy, regardless of the veneering technique. In 3Y‐TZP, a wider range of elemental interdiffusion was observed in the pressing technique than in the layering technique.

**Conclusions:**

In bilayered restorations with a 3Y‐TZP substructure, the pressing technique yielded higher bonding strength than layering. Using the layering technique, 3Y‐TZP showed a lower SBS than the Co‐Cr alloy. In bilayered restorations using 3Y‐TZP as a substructure, the veneering technique and thermal compatibility of the materials must be considered.

Bilayered restorations, combining a substructure for mechanical properties and a veneering material to meet esthetic demands, are widely used. Successful bilayered restorations should have good mechanical properties in both the substructure and veneering material and a shear bond strength (SBS) greater than 25 MPa between the two materials.^1^, ^2^, ^3^ Metal (cobalt‐chromium [Co‐Cr] alloy)‐ceramic restorations and zirconia (3 mol% yttrium‐stabilized tetragonal zirconia polycrystal [3Y‐TZP])‐ceramic restorations are the most common bilayered restorations. The bonding mechanism in metal‐ceramics is categorized into chemical bonding, mechanical interlocking, van der Waals forces, and compressive forces.^1^, ^2^, ^4^, ^5^, ^6^ However, in zirconia ceramics, the bonding mechanism between the 3Y‐TZP substructure and the veneering ceramic is debatable. Some studies have shown that the interdiffusion of elements demonstrating chemical bonds in veneering ceramic diffuses to a depth of 5–10 µm into the 3Y‐TZP surface.^7^, ^8^, ^9^ However, other studies reported that elemental interdiffusion does not occur between zirconia and feldspathic porcelain.^10^ Qualitative analysis of the interface does not reveal any chemical reaction layer, indicating the absence of chemical bonding.^11^, ^12^ Thus, in zirconia‐ceramic bonding, the wettability of the veneering ceramic on the 3Y‐TZP substructure and the resulting micromechanical interlocking are the most important bonding mechanisms between the two materials.^13^, ^14^

The compressive force is caused by the difference in the coefficients of thermal expansion (CTE) between the substructure and veneering material. When the CTE of the Co‐Cr alloy is higher than that of the ceramic, internal compressive stress is generated, which is beneficial for metal‐ceramic bonding.^2^ However, Asaoka and Teck^15^ reported that the main cause of ceramic fracture is when the difference of the CTE in metal‐ceramic restorations is greater than 2 × 10^−6^ K^−1^. Thermal compatibility in bilayered restorations is achieved when the expansion or contraction caused by temperature changes during the firing of the veneering ceramic is within a range similar to that of the substructure.^4^, ^16^ Additionally, thermal compatibility is an important factor determining the reliability of zirconia‐ceramic restoration.^17^, ^18^, ^19^ Due to the unfavorable thermal compatibility between 3Y‐TZP and feldspathic porcelain, zirconia‐ceramic restorations have been reported to cause more frequent chipping than metal‐ceramic restorations.^20^ Nevertheless, studies on adhesive strength according to veneering and substructure materials considering thermal compatibility are rare.

Veneering bilayered restorations involves both layering and pressing techniques.^1^, ^21^, ^22^ The layering technique can reproduce high‐level esthetics by considering tooth structure; however, the layering technique is technique‐sensitive.^23^ In contrast, the pressing technique uses the lost‐wax technique and injects the heated glass ceramic into a mold.^1^, ^21^ This method is less technique‐sensitive compared to the layering technique.^1^, ^22^, ^24^, ^25^, ^26^ Moreover, the ceramic used in the pressing technique is a particle‐reinforced glass ceramic, exhibiting superior mechanical strength than feldspathic porcelain, primarily utilized in the layering technique.^27^ However, differences in the veneering materials result in different thermal compatibilities. Additionally, the heat‐treatment temperature and procedure vary depending on the veneering technique used.^17^, ^28^, ^29^, ^30^, ^31^ Based on these factors, the thermal compatibility between the substructure and veneering material can vary depending on the veneering technique.^4^, ^32^, ^33^

In bilayered restorations, including metal‐ceramic or zirconia‐ceramic, evaluating the bonding strength according to the materials and the veneering techniques is necessary. This study aimed to evaluate the SBS according to the substructure materials (Co‐Cr alloy and 3Y‐TZP) and veneering techniques (layering and pressing). The null hypothesis posited that the SBS between the substructure and veneering ceramic would not vary based on the material of the substructure or the ceramic veneering technique.

## MATERIALS AND METHODS

The substructure had a cuboid shape (15 × 15 × 5 mm^3^), and the veneering ceramic was disk‐shaped with a 5‐mm diameter and 2‐mm thickness. The veneering ceramic was designed to be built at the center of the upper surface of the substructure. The test group was divided into four groups (*n* = 15 per group) according to the substructure material and the ceramic veneering technique (Table 1). Each group required 15 specimens for the SBS test, as determined using G*Power (version 3.1.9.6, Kiel University, Kiel, Germany). To consistently replicate the dimensions of the designed Co‐Cr alloy substructure, molds were made using a three‐dimensional (3D) printer (Dental 3DPrinter‐P, Dentium Co., Seoul, South Korea) and a transparent light‐polymerizing resin (DG‐1, Veltz 3D Co., Incheon, South Korea). A wax pattern was formed by melting and injecting inlay wax (Maves Inlay Wax, Maves Co., Cleveland, OH, USA) into a mold. Casting was performed using the lost‐wax technique via spruing and investing (BC‐VEST CB‐700, Bukwang Inc., Busan, South Korea). A Co‐Cr alloy substructure was cast by melting and injecting a Co‐Cr ingot into the fabricated mold using a centrifugal casting machine (Casting Machine, Osung M&D, Gimpo, South Korea). After casting, air‐particle abrasion was performed by spraying alumina (Al_2_O_3_) with a particle size of 250 µm at a pressure of 3–4 bar on the surface of the area where ceramic was to be veneered. The opaque porcelain (Noritake Super Porcelain EX‐3—Powder Opaque with Opaque liquid, Kuraray Noritake Dental Inc., Tokyo, Japan) was applied thinly (thickness: 0.1 mm or less), followed by primary firing (Table 2—Schedule: 1st opaque). The same opaque porcelains with a thickness of approximately 0.3 mm were applied again, followed by a secondary firing (Table 2—Schedule: 2nd opaque). A stereolithography (STL) file consistent with the dimensions of the designed 3Y‐TZP substructure was generated using a computer‐aided design (CAD) program (Shapr3D CAD modeling, Shapr3D Zrt, Budapest, Hungary). Using this STL file, the pre‐sintered 3Y‐TZP disk (ZirtoothTM, HASS Co., Gangneung, South Korea) was milled using a machine (rainbow Mill‐Zr 2nd, Dentium, Seoul, South Korea). The milled specimens were sintered in a furnace (Duotronpro S‐600, ADD‐IN Co., Goyang, South Korea) according to the manufacturer's recommended temperature schedule. On the substructure of the feldspathic porcelain layered on Co‐Cr alloy (FLM) and feldspathic porcelain layered on 3Y‐TZP (FLZ) groups, body porcelain (Noritake Super Porcelain EX‐3 – Body with Forming liquid, Kuraray Noritake Dental Inc., Tokyo, Japan) was built, which was approximately 0.8 mm thicker than the designed dimension (2‐mm thickness), and then fired (Table 2—Schedule: Body porcelain). After the specimen was cooled to room temperature, the porcelain was finished and polished based on the designed dimensions using a rotary instrument with a diamond bur (Dura‐Green DIA, Shofu, Kyoto, Japan) and a silicon point (Ceramisté, Shofu). The fabricated wax pattern for the pressing technique attached to the substructure according to the specimen design was sprued and invested (Amber Vest, HASS Co., Gangneung, South Korea). The investment ring was preheated to 860°C for 30 min in the furnace to burn out the wax pattern, and the glass ceramic ingot was pressed into the mold using a heat‐press furnace (Rosetta Press, HASS Co., Gangneung, South Korea) (Table 2—Schedule: Pressing tech.). The mold was cooled to room temperature, and the surface was treated with an airborne particle abrasion with 50 µm Al_2_O_3_ particles at a pressure of 3–4 bar. All the specimens were washed using an ultrasonic cleaner in distilled water for 10 min and then dried by spraying air for 10 s after cleaning. After storage in distilled water at 37°C for 24 h, all specimens were thermocycled between 5°C and 55°C with a dwell time of 2 s (Thermal Cyclic Tester, R&B Inc, Daejeon, South Korea) for 10,000 cycles to simulate 1 year. All specimens were stored in distilled water at 37°C for 24 h.

**TABLE 1:** Specimen group and materials used in the study with their thermal expansion coefficients.

Group		Material	CTE (× 10^−6^ K^−1^)
FLM	Veneered ceramic	Feldspathic porcelain (Noritake Super Porcelain EX‐3, Kuraray Noritake Dental Inc., Tokyo, Japan)	12.4
	Substructure material	Co‐Cr base‐metal alloy (StarLoy C, Dentsply Sirona, PA, Germany)	14.0
LPM	Veneered ceramic	Lithium silicate (Amber LiSi‐POM, HASS Co., Gangneung, South Korea)	12.0
	Substructure material	Co‐Cr base‐metal alloy (StarLoy C, Dentsply Sirona, PA, Germany)	14.0
FLZ	Veneered ceramic	Feldspathic porcelain (Noritake Super Porcelain EX‐3, Kuraray Noritake Dental Inc., Tokyo, Japan)	12.4
	Substructure material	3 mol% yttria‐stabilized tetragonal zirconia polycrystal (Zirtooth, HASS Co., Gangneung, South Korea)	10.5
LPZ	Veneered ceramic	Lithium silicate (Amber LiSi‐POZ, HASS Co., Gangneung, South Korea)	9.6
	Substructure material	3 mol% yttria‐stabilized tetragonal zirconia polycrystal (Zirtooth, HASS Co., Gangneung, South Korea)	10.5

Abbreviations: Co‐Cr, cobalt‐chromium; FLM, feldspathic porcelain layered on Co‐Cr alloy; FLZ, feldspathic porcelain layered on 3Y‐TZP; LPM, lithium silicate pressed on Co‐Cr alloy; LPZ, lithium disilicate pressed on 3Y‐TZP; CTE, coefficients of thermal expansion

**TABLE 2:** Sintering schedule of ceramic materials.

Layering technique	Starting temp. (°C)	Start vacuum (°C)	Heating rate (°C/min)	Holding time (min)	Release vacuum (°C)	Max temp. (°C)
1st opaque	650	650	55	4	950	960
2nd opaque	650	650	55	4	950	960
Body porcelain	600	600	45	2	920	930
Pressing technique	Starting temp. (°C)	Start vacuum (°C)	Heating rate (°C/min)	Holding time (min)	Release vacuum (°C)	Max temp. (°C)
Amber LiSi‐POM	700	700	60	15	820	820
Amber LiSi‐POZ	700	700	45	15	915	915

Abbreviation: Temp., Temperature.

Each specimen was mounted on a stainless‐steel mounting jig with the interface between the substructure of the specimen and the veneering ceramic positioned parallel to the outer surface of the jig. Shear stress was applied horizontally to the interface between the substructure and veneering ceramic using a universal testing machine (High Force Universal Testing Machines: 5982, Instron, MA, USA). The load (Newton; N) was measured and recorded as the veneering ceramic collapsed. The SBS (MPa) was calculated by dividing the measured load by the interface area (mm^2^). After performing the SBS test on all the specimens, the failure mode was observed and classified as follows: adhesive failure (complete separation of the veneering ceramic from the substructure of the specimen), cohesive failure (failure within the veneering ceramic), and mixed failure (mixture of adhesive and cohesive failure). Specimens representative of each group were selected from the specimens that completed the SBS test. An Au‐Pb sputter ion coating was applied to the fracture surface of the substructure side and the veneering ceramic side of the specimens. The shape of the fracture surface was observed using the Fe‐scanning electron microscope (Quanta FEG 250, FEI, Hillsboro, OR, USA).

For interface analysis, one specimen was fabricated for each group. Each specimen was vertically cut using a cutting device (Secotom‐50, Struers, Ballerup, Denmark) to cross the substructure and the veneering ceramic together (3500 rpm, feed speed 0.115 mm/s). The cut specimens were washed for 60 s with 99.9% anhydrous ethyl alcohol using an ultrasonic cleaner (UCP‐10; Jeio Tech, Daejeon, South Korea). An Au‐Pd sputter ion coating was applied to the cut surface, and the elemental distribution at the interface was evaluated using EDS (Apollo XP, EDAX, Mahwah, NJ, USA).

Statistical analysis of the results was performed using SPSS software (SPSS Statistics 23.0, IBM Corporation, Chicago, IL, USA). Normality was evaluated using the Shapiro‐Wilk test and homogeneity was assessed using Levene's test. The difference in SBS was analyzed using a two‐way analysis of variance based on the substructure material, and the ceramic veneering technique was analyzed. A post‐hoc test was conducted using Tukey's honestly significant difference test to determine whether the SBS showed a statistically significant difference. Statistical significance was analyzed at a confidence interval of 95%.

## RESULTS

Table 3 displays the significant interaction between the substructure material and the veneering technique. Considering substructure materials, no significant difference was found between the SBS of the Co‐Cr alloy (28.8 ± 6.1 MPa) and 3Y‐TZP (32.5 ± 14.4 MPa). However, in the veneering technique, the pressing (34.9 ± 14.3 MPa) had a higher SBS than the layering (25.7 ± 7.3 MPa). The SBS test results for each group are presented in Table 4. The lithium disilicate pressed on 3Y‐TZP (LPZ) group (43.4 ± 11.9 MPa) yielded the highest SBS, followed by the FLM (29.7 ± 6.6 MPa), lithium silicate pressed on Co‐Cr alloy (LPM) (28.0 ± 5.7 MPa), and FLZ groups (21.7 ± 5.7 MPa). Tukey's honestly significant difference test results revealed that the SBS of the LPZ group was significantly different from that of all other groups. A significant difference was also found between the SBS of the FLM and FLZ groups.

**TABLE 3:** Two‐way ANOVA results on the effects of substructure or veneering technique, and their interaction on the shear bond strength (SBS).

		Sum of square	Degree of freedom	*p*‐value
Shear bond strength	Substructure material	205.668	1	0.076
	Veneering technique	1504.118	1	<0.001
	Substructure material * veneering technique	2062.092	1	<0.001
	Error	3511.673	56	
	Total	63,760.576	60	
	Corrected total	7283.551	59	

**TABLE 4:** Shear bond strength (SBS) values.

Group	SBS (MPa)
FLM	29.7 ± 6.6^b^
LPM	28.0 ± 5.7^bc^
FLZ	21.7 ± 5.7^c^
LPZ	43.4 ± 11.9^a^

*Note*: Different superscript letters indicate statistically significant differences (*p* < 0.05).

Abbreviations: FLM, feldspathic porcelain layered on Co‐Cr alloy; FLZ, feldspathic porcelain layered on 3Y‐TZP; LPM, lithium silicate pressed on Co‐Cr alloy; LPZ, lithium disilicate pressed on 3Y‐TZP.

**FIGURE 1:**
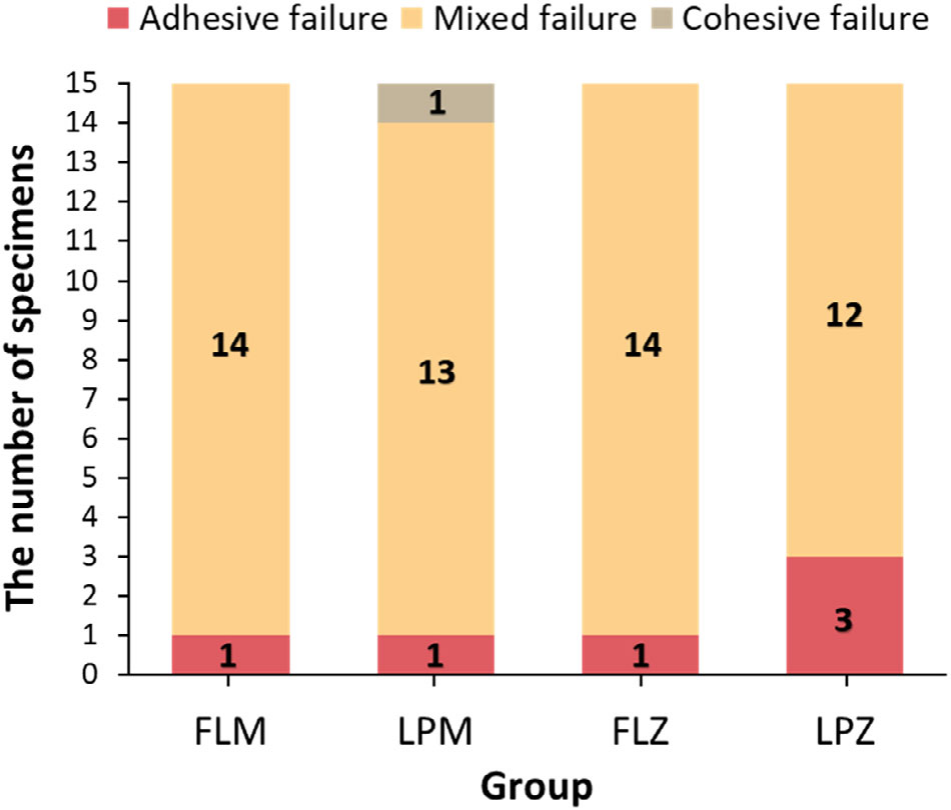
Number of failure modes. FLM, feldspathic porcelain layered on Co‐Cr alloy; FLZ, feldspathic porcelain layered on 3Y‐TZP; LPM, lithium silicate pressed on Co‐Cr alloy; LPZ, lithium disilicate pressed on 3Y‐TZP. The number written inside the bar graph indicates the number of specimens.

The failure modes of all the groups are presented in Figure 1. Mixed failure was the most frequent failure in all groups. Cohesive failure was observed solely in the LPM group, whereas the LPZ group exhibited adhesive failure in three cases, and the other groups had only one instance of adhesive failure.

**FIGURE 2:**
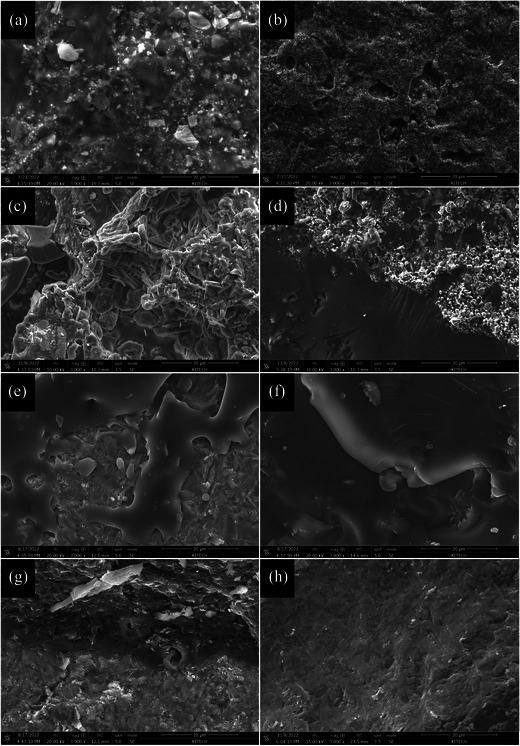
Representative SEM images of the fracture surface of the specimen in each group (magnification x 3000). (a) FLM group, Co‐Cr alloy side, (b) FLM group, veneer side, (c). LPM group, Co‐Cr alloy side, (d) LPM group, veneer side, (e) FLZ group, 3Y‐TZP side, (f) FLZ group, veneer side, (g) LPZ group, 3Y‐TZP side, and (h) LPZ group, veneer side. 3Y‐TZP, 3 mol% yttrium‐stabilized tetragonal zirconia polycrystal; Co‐Cr, cobalt‐chromium; FLM, feldspathic porcelain layered on Co‐Cr alloy; FLZ, feldspathic porcelain layered on 3Y‐TZP; LPM, lithium silicate pressed on Co‐Cr alloy; LPZ, lithium disilicate pressed on 3Y‐TZP; SEM, scanning electron microscope.

Scanning electron microscope (SEM) images of the fracture surfaces in each group are shown in Figure 2. On the fracture surface of the FLM specimen, a glass matrix and inorganic fillers were observed, along with residual opaque porcelain (Figure 2a,b). On the Co‐Cr alloy side of the LPM specimen, lithium disilicate crystals were observed among the remaining opaque porcelain (Figure 2c). On the veneer side, a mixed matrix of glass ceramic and opaque porcelain was observed (Figure 2d). On the 3Y‐TZP side of the FLZ specimen, poor wetting of the feldspathic porcelain against the polycrystalline phase of 3Y‐TZP was observed (Figure 2e), whereas only the feldspathic porcelain glass matrix was observed on the veneer side (Figure 2f). On the 3Y‐TZP side of the LPZ specimen, good wettability of LS2 glass ceramic against the polycrystalline phase of 3Y‐TZP was observed (Figure 2g), and only the matrix of lithium disilicate glass ceramic was observed on the veneer side (Figure 2h).

**FIGURE 3:**
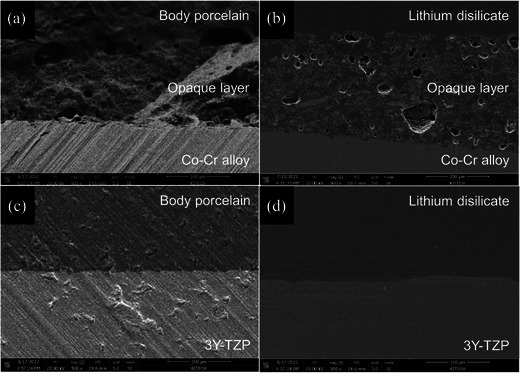
SEM images on the interface of the substructure and the veneer material (magnification x 500). (a) FLM group, (b) LPM group, (c) FLZ group, and (d) LPZ group. 3Y‐TZP, 3 mol% yttrium‐stabilized tetragonal zirconia polycrystal; Co‐Cr, cobalt‐chromium; FLM, feldspathic porcelain layered on Co‐Cr alloy; FLZ, feldspathic porcelain layered on 3Y‐TZP; LPM, lithium silicate pressed on Co‐Cr alloy; LPZ, lithium disilicate pressed on 3Y‐TZP; SEM, scanning electron microscope.

**FIGURE 4:**
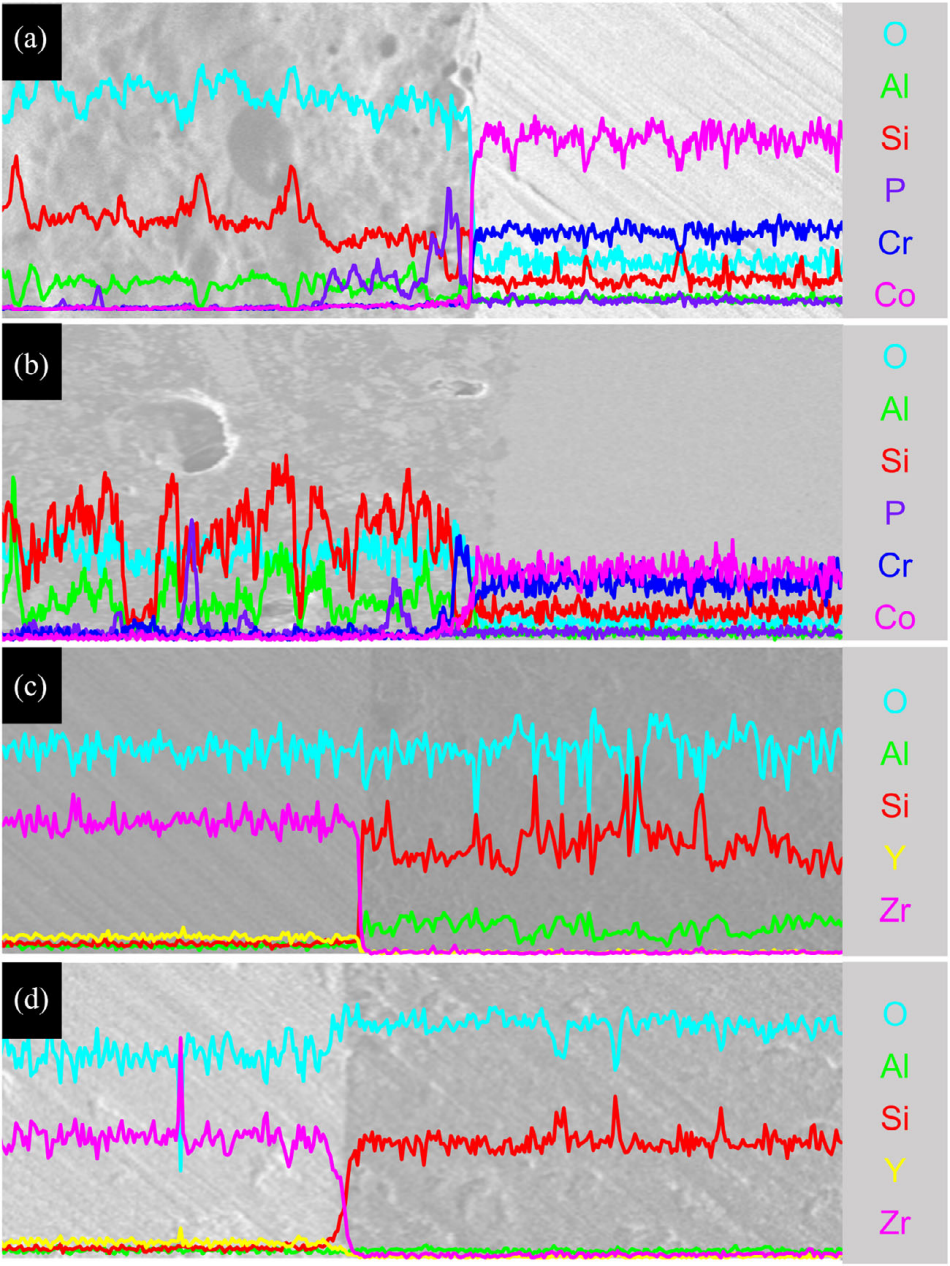
Energy‐dispersive spectroscopy (EDS) linear analysis on the interface of the substructure and veneer material. (a) FLM group, (b) LPM group, (c) FLZ group, and (d) LPZ group. FLM, feldspathic porcelain layered on Co‐Cr alloy; FLZ, feldspathic porcelain layered on 3Y‐TZP; LPM, lithium silicate pressed on Co‐Cr alloy; LPZ, lithium disilicate pressed on 3Y‐TZP.

In the SEM images, pores in the opaque layer were observed at the interface of the LPM and FLM specimens, along with natural transitions of the opaque layer and veneering ceramic (Figure 3a,b). At the interface in the FLZ and LPZ specimens, 3Y‐TZP and the veneering ceramic were clearly distinguished (Figure 3c,d). In the FLM and LPM groups, sufficient elemental interdiffusion of the veneering ceramic through the opaque layer was observed in the energy‐dispersive spectroscopy (EDS) linear analysis (Figure 4a,b). In the FLZ and LPZ groups, the elemental distribution graph also showed a monotonous pattern (Figure 4c,d).

## DISCUSSION

The SBS was not significantly affected by the substructure material; however, the veneering technique had a significant effect on the SBS. Therefore, the null hypothesis was partially rejected. An interaction between two factors (substructure material and veneering technique) was observed. The veneering technique had a greater impact on 3Y‐TZP than on the Co‐Cr alloy substructure.

In the Co‐Cr alloy substructure groups, the FLM group exhibited better thermal compatibility than the LPM group in this study. The CTE difference in the FLM group (1.6 × 10^−6^ K^−1^) was relatively closer to the ideal value (0.5–1.0 × 10^−6^ K^−1^) than the CTE difference in the LPM group (2.0 × 10^−6^ K^−1^).^16^ However, consistent with previous studies,^21^, ^34^, ^35^ the FLM and LPM groups showed no significant differences in the SBS because direct bonding between the veneering ceramic and Co‐Cr alloy occurred in the opaque layer. Good wettability was observed in the fracture surface and interfacial SEM image analysis of the LPM specimen, indicating the mechanical bonding of the lithium silicate glass ceramic to the opaque layer. EDS linear analysis showed sufficient element interdiffusion, indicating chemical bonding between the lithium silicate glass ceramic and the opaque layer. Moreover, the mechanical properties of the veneering ceramic may have influenced the SBS results, which may have been advantageous in the LPM group with better mechanical properties of the veneering ceramic.^36^, ^37^, ^38^ These factors are presumed to compensate for the distinctions between the pressing and layering techniques of the Co‐Cr alloy.

When the substructure was 3Y‐TZP, the LPZ group had a higher SBS than the FLZ group, which is consistent with findings from previous studies.^35^, ^39^ However, conflicting results have been reported in other studies.^31^, ^40^, ^41^ The bonding mechanism between 3Y‐TZP and veneering ceramics remains unclear and has not been precisely identified yet.^30^, ^31^, ^35^, ^39^, ^40^, ^41^, ^42^ This is because the type of material used and the specific procedure for ceramic veneering led to differences in the results among the studies. In this study, the difference in CTE between 3Y‐TZP and the veneering ceramic in the FLZ group (‐1.9 × 10^−6^ K^−1^) indicated a large thermal incompatibility; however, the difference in CTE in the LPZ group (0.9 × 10^−6^ K^−1^) was within the ideal range. Additionally, the wettability of the veneering ceramic on 3Y‐TZP was better in the LPZ group than in the FLZ group, as observed in the SEM image of the fracture surface. Moreover, EDS linear analysis revealed a broader interdiffusion range of elements in the LPZ group compared to the FLZ group. Overall, the LPZ group exhibited superior thermal compatibility between 3Y‐TZP and the veneering ceramic, alongside enhanced mechanical and chemical bonding, compared to the FLZ group.

The impact of the veneering technique on SBS was more pronounced when the substructure was 3Y‐TZP instead of Co‐Cr. This was attributed to differences in thermal compatibility between the substructure and the veneering material. The difference in thermal compatibility was greater for 3Y‐TZP than for Co‐Cr. In addition, the Co‐Cr substructure was not directly bonded to the veneering material. Instead, the Co‐Cr substructure and veneering material were bonded to the opaque layer, composed of a feldspar‐based ceramic. Therefore, the thermal compatibility effect may have been offset by the opaque layer. The effect of an opaque layer on thermal compatibility necessitates further studies.

As shown in the SEM images of the interface, more pores were found inside the opaque layer in the LPM group than in the FLM group. Pores were generated when an opaque layer was fired on the Co‐Cr alloy or when the invested Co‐Cr alloy was exposed to high temperatures during pressing.^43^, ^44^ These act as weak areas when shear stress is applied.^21^ Conversely, the LPZ group would have been relatively less vulnerable to SBS due to fewer defects at the interface between 3Y‐TZP and the veneering ceramic.^21^ Moreover, the better compatibility of the LPZ group had a favorable effect on the bonding strength because a good degree of compressive stress was formed between the substructure and the veneer material.^17^, ^18^, ^19^ Some studies have demonstrated that when a glass ceramic is pressed, the low viscosity and excellent wettability of the glass phase to 3Y‐TZP can penetrate grain boundaries.^9^ Altogether, the favorable thermal compatibility and excellent micromechanical bonding in the pressing technique were assumed to cause the high bond strength between the substructure and the veneering ceramic in the LPZ group in this study, even if the possibility of chemical bonding between the two materials was excluded.

The most common failure mode observed in all groups was mixed failure. Mixed failure occurred because the bond strength between the two materials was sufficient, comparable to the fracture strength of the veneering material.^45^ Cohesive failure occurred only in the LPM group, where the bond strength exceeded the fracture strength of both the opaque layer and the veneering ceramic.^46^ This type of failure was likely within the opaque layer, which is a feldspar‐based ceramic. This failure was attributed to the superior mechanical properties of lithium disilicate, a veneering material in the LPM group, compared to those of the feldspar‐based ceramic.^47^ Adhesive failure occurred only in the LPZ group, indicating that the adhesive area was the weakest point. This can occur because of the superior mechanical strength of the veneering materials.^48^ In the LPZ group, the veneering material was lithium disilicate, which has a high fracture strength and was bonded directly to the substructure. The high fracture strength of the veneering material made the adhesive area the weakest point. Otherwise, the LPZ group exhibited the highest SBS and the most appropriate thermal compatibility. Therefore, interpreting the failure mode in the LPZ group requires caution due to potential misinterpretation.

A limitation of this study was the inclusion of variations in veneering techniques, which also involved differences in veneering materials. These distinctions might have influenced the SBS results due to variations in the mechanical properties of the veneering ceramics. However, given the absence of a significant difference in the failure mode analysis, such influences were not expected to substantially impact the study's outcomes. Moreover, this study was meaningful as it assessed clinically applicable materials.

## CONCLUSION

In this study, no significant difference was observed in the SBS between the layering and pressing techniques when the substructure was a Co‐Cr alloy. However, with a substructure of 3Y‐TZP, the pressing technique yielded a higher SBS than the layering technique. Moreover, in cases where the layering technique was employed, 3Y‐TZP exhibited a lower SBS than the Co‐Cr alloy. Thus, in bilayered restorations using 3Y‐TZP as a substructure, the veneering technique and thermal compatibility of the materials should be considered more than in Co‐Cr substructures.

## CONFLICT OF INTEREST STATEMENT

The authors deny any conflicts of interest related to this study.

## References

[1] Galiatsatos P , Galiatsatos A , Phillipatos G . Characterization of the interface of heat‐pressed glass‐ceramic masses on metal support Cr‐Co in metal‐ceramic prosthetic restorations. J Contemp Dent Pract. 2021;22(4):335–341. 34266999

[2] Sipahi C , Ozcan M . Interfacial shear bond strength between different base metal alloys and five low fusing feldspathic ceramic systems. Dent Mater J. 2012;31(3):333–337. 22673457

[3] ISO . Dentistry—compatibility testing for metal‐ceramic and ceramic‐ceramic systems. International Organization for Standardization.ISO 9693:2019.

[4] Bagby M , Marshall S , Marshall G Jr . Metal ceramic compatibility: a review of the literature. J Prosthet Dent. 1990;63(1):21–25. 2404102 10.1016/0022-3913(90)90259-f

[5] King BW , Tripp H , Duckworth WH . Nature of adherence of porcelain enamels to metals. J Am Ceram Soc. 1959;42(11):504–525.

[6] Yamamoto M . Metal‐ceramics: principle and methods of Makoto Yamamoto. Quintessence Pub. 1985:268–302.

[7] Aboushelib MN , Kleverlaan CJ , Feilzer AJ . Microtensile bond strength of different components of core veneered all‐ceramic restorations: part II: zirconia veneering ceramics. Dent Mater. 2006;22(9):857–863. 16376981 10.1016/j.dental.2005.11.014

[8] Yin JY , Zhang ZT , Ai HJ , Si WJ , Bao Y . Study on the compatibility of yttria‐stabilized zirconia framework bonded to the corresponding veneering ceramic. China J Stomatol 2009;27(6):669–672. 20077908

[9] Choi JE , Waddell JN , Torr B , Swain MV . Pressed ceramics onto zirconia. Part 1: comparison of crystalline phases present, adhesion to a zirconia system, and flexural strength. Dent Mater. 2011;27(12):1204–1212. 21958727 10.1016/j.dental.2011.08.006

[10] Kawai Y , Uo M , Watari F . Microstructure evaluation of the interface between dental zirconia ceramics and veneering porcelain. Nano Biomedicine. 2010;2(1):31–36.

[11] Ban S . Reliability and properties of core materials for all‐ceramic dental restorations. Jpn Dent Sci Rev. 2008;44(1):3–21.

[12] Kim S , Cho H , Lee Y , Choi S , Moon H . Bond strength of Y‐TZP–zirconia ceramics subjected to various surface roughening methods and layering porcelain. Surf Interface Anal. 2010;42(6‐7):576–580.

[13] Çömlekoğlu ME , Dündar M , Özcan M , Güngör MA , Gökçe B , Artunç C . Evaluation of bond strength of various margin ceramics to a zirconia ceramic. J Dent. 2008;36(10):822–827. 18620791 10.1016/j.jdent.2008.05.019

[14] Liu D , Matinlinna JP , Pow EH . Insights into porcelain to zirconia bonding. J Adhes Sci Technol. 2012;26(8‐9):1249–1265.

[15] Asaoka K , Tesk JA . Transient and residual stress in a porcelain‐metal strip. J Dent Res. 1990;69(2):463–469. 2307748 10.1177/00220345900690020901

[16] Wataha JC . Alloys for prosthodontic restorations. J Prosthet Dent. 2002;87(4):351–363. 12011845 10.1067/mpr.2002.123817

[17] Swain M . Unstable cracking (chipping) of veneering porcelain on all‐ceramic dental crowns and fixed partial dentures. Acta Biomater. 2009;5(5):1668–1677. 19201268 10.1016/j.actbio.2008.12.016

[18] Guazzato M , Walton T , Franklin W , Davis G , Bohl C , Klineberg I . Influence of thickness and cooling rate on development of spontaneous cracks in porcelain/zirconia structures. Aust Dent J. 2010;55(3):306–310. 20887519 10.1111/j.1834-7819.2010.01239.x

[19] Guess PC , Kuliš A , Witkowski S , Wolkewitz M , Zhang Y , Strub JR . Shear bond strengths between different zirconia cores and veneering ceramics and their susceptibility to thermocycling. Dent Mater. 2008;24(11):1556–1567. 18466964 10.1016/j.dental.2008.03.028

[20] Sailer I , Gottner J , Känel S , Franz Hämmerle CH . Randomized controlled clinical trial of zirconia‐ceramic and metal‐ceramic posterior fixed dental prostheses: a 3‐year follow‐up. Int J Prosthodont. 2009;22(6):553–560. 19918588

[21] Schweitzer DM , Goldstein GR , Ricci JL , Silva NR , Hittelman EL . Comparison of bond strength of a pressed ceramic fused to metal versus feldspathic porcelain fused to metal. J Prosthodont. 2005;14(4):239–247. 16359480 10.1111/j.1532-849X.2005.00052.x

[22] Lee J‐H . An accelerated technique for a ceramic‐pressed‐to‐metal restoration with CAD/CAM technology. J Prosthet Dent. 2014;112(5):1021–1023. 24952883 10.1016/j.prosdent.2014.05.015

[23] Zarone F , Russo S , Sorrentino R . From porcelain‐fused‐to‐metal to zirconia: clinical and experimental considerations. Dent Mater. 2011;27(1):83–96. 21094996 10.1016/j.dental.2010.10.024

[24] Fahmy NZ , Salah E . An in vitro assessment of a ceramic‐pressed‐to‐metal system as an alternative to conventional metal ceramic systems. J Prosthodont. 2011;20(8):621–627. 22182223 10.1111/j.1532-849X.2011.00767.x

[25] Goldin EB , Boyd NW III , Goldstein GR , Hittelman EL , Thompson VP . Marginal fit of leucite‐glass pressable ceramic restorations and ceramic‐pressed‐to‐metal restorations. J Prosthet Dent. 2005;93(2):143–147. 15674224 10.1016/j.prosdent.2004.10.023

[26] Bayramoğlu E , Özkan YK , Yildiz C . Comparison of marginal and internal fit of press‐on‐metal and conventional ceramic systems for three‐and four‐unit implant‐supported partial fixed dental prostheses: an in vitro study. J Prosthet Dent. 2015;114(1):52–58. 25858218 10.1016/j.prosdent.2015.01.002

[27] Kelly JR . Dental ceramics: current thinking and trends. Dental Clinics. 2004;48(2):513–530. 10.1016/j.cden.2004.01.003 15172614

[28] Schweitzer DM , Goldstein GR , Ricci JL , Silva N , Hittelman EL . Comparison of bond strength of a pressed ceramic fused to metal versus feldspathic porcelain fused to metal. J Prosthodont. 2005;14(4):239–247. 16359480 10.1111/j.1532-849X.2005.00052.x

[29] Farzin M , Khaledi AA , Malekpour B , Naseri MH . Evaluation of bond strength of pressed and layered veneering ceramics to nickel‐chromium alloy. J Dent (Shiraz). 2015;16(3 Suppl):230–236. 26535402 PMC4623836

[30] Zaher AM , Hochstedler J , Rueggeberg FA , Kee EL . Shear bond strength of zirconia‐based ceramics veneered with 2 different techniques. J Prosthet Dent. 2017;118(2):221–227. 28222866 10.1016/j.prosdent.2016.11.016

[31] Pharr SW , Teixeira EC , Verrett R , Piascik JR . Influence of veneering fabrication techniques and gas‐phase fluorination on bond strength between zirconia and veneering ceramics. J Prosthodont. 2016;25(6):478–484. 26849102 10.1111/jopr.12451

[32] Fairhurst CW , Anusavice KJ , Hashinger DT , Ringle RD , Warren Twiggs S . Thermal expansion of dental alloys and porcelains. J Biomed Mater Res. 1980;14(4):435–446. 6995461 10.1002/jbm.820140410

[33] Alpízar M , Castillo R , Chinè B . Thermal stress analysis by finite elements of a metal‐ceramic dental bridge during the cooling phase of a glaze treatment. J Mech Behav Biomed Mater. 2020;104:103661. 32174418 10.1016/j.jmbbm.2020.103661

[34] Khmaj MR , Khmaj AB , Brantley WA , Johnston WM , Dasgupta T . Comparison of the metal‐to‐ceramic bond strengths of four noble alloys with press‐on‐metal and conventional porcelain layering techniques. J Prosthet Dent. 2014;112(5):1194–1200. 25134992 10.1016/j.prosdent.2014.06.004

[35] Ishibe M , Raigrodski AJ , Flinn BD , Chung K‐H , Spiekerman C , Winter RR . Shear bond strengths of pressed and layered veneering ceramics to high‐noble alloy and zirconia cores. J Prosthet Dent. 2011;106(1):29–37. 21723991 10.1016/S0022-3913(11)60090-5

[36] Simba BG , Ribeiro MV , Suzuki PA , Alves MFR , Strecker K, Santos CD . Mechanical properties of lithium metasilicate after short‐term thermal treatments. J Mech Behav Biomed Mater. 2019;98:179–186. 31247537 10.1016/j.jmbbm.2019.06.011

[37] Soares VO , Serbena FC , Mathias I , Crovace MC , Zanotto ED . New, tough and strong lithium metasilicate dental glass‐ceramic. Ceram Int. 2021;47(2):2793–2801.

[38] Seghi RR , Denry IL , Rosenstiel SF . Relative fracture toughness and hardness of new dental ceramics. J Prosthet Dent. 1995;74(2):145–150. 8537920 10.1016/s0022-3913(05)80177-5

[39] Subash M , Vijitha D , Deb S , Satish A , Mahendirakumar N . Evaluation of shear bond strength between zirconia core and ceramic veneers fabricated by pressing and layering techniques: in vitro study. J Pharm Bioallied Sci. 2015;7(Suppl 2):S612–S615. 26538929 10.4103/0975-7406.163568 PMC4606671

[40] Sim JY , Lee WS , Kim JH , Kim HY , Kim WC . Evaluation of shear bond strength of veneering ceramics and zirconia fabricated by the digital veneering method. J Prosthodont Res. 2016;60(2):106–113. 26679601 10.1016/j.jpor.2015.11.001

[41] Kanat B , Çömlekoğlu EM , Dündar‐Çömlekoğlu M , Hakan Sen B , Özcan M , Ali Güngör M . Effect of various veneering techniques on mechanical strength of computer‐controlled zirconia framework designs. J Prosthodont. 2014;23(6):445–455. 24417370 10.1111/jopr.12130

[42] Alghazzawi TF , Janowski GM . Effect of liner and porcelain application on zirconia surface structure and composition. Int J Oral Sci. 2016;8(3):164–171. 27445089 10.1038/ijos.2016.20 PMC5113090

[43] Yoshimura HN , Da Cruz AC , Zhou Y , Tanaka H . Sintering of 6H(α)‐SiC and 3C(β)‐SiC powders with B_4_C and C additives. J Mater Sci. 2002;37:1541–1546.

[44] Sarna‐Boś K , Skic K , Sobieszczański J , Boguta P , Chałas R . Contemporary approach to the porosity of dental materials and methods of its measurement. Int J Mol Sci. 2021;22(16):8903. 34445606 10.3390/ijms22168903 PMC8396236

[45] Muñoz MA , Baggio R , Mendes YBE , Gomes GM , Luque‐Martinez I , Loguercio AD , et al. The effect of the loading method and cross‐head speed on resin–dentin microshear bond strength. Int J Adhes Adhes. 2014;50:136–141.

[46] Sano H , Shono T , Sonoda H , Takatsu T , Ciucchi B , Carvalho R , et al. Relationship between surface area for adhesion and tensile bond strength—evaluation of a micro‐tensile bond test. Dent Mater. 1994;10(4):236–240. 7664990 10.1016/0109-5641(94)90067-1

[47] Zhang Y , Kelly JR . Dental ceramics for restoration and metal veneering. Dental Clinics. 2017;61(4):797–819. 28886769 10.1016/j.cden.2017.06.005 PMC5657342

[48] Nogueira VF , Rodrigues CDS , Grangeiro MTV , Contreras LPC , Marinho RMM , Bottino MA . Interface adhesion on layered zirconia: effects of the veneering ceramic material and veneering technique. J Prosthodont 2023;1–7. 10.1111/jopr.13756 37626443

